# Dealing with ethical challenges: a focus group study with professionals in mental health care

**DOI:** 10.1186/1472-6939-16-4

**Published:** 2015-01-16

**Authors:** Bert Molewijk, Marit Helene Hem, Reidar Pedersen

**Affiliations:** Centre for Medical Ethics, Institute of Health and Society, Faculty of Medicine, University of Oslo, P.O. Box 1130, Blindern NO-0318 Oslo, Norway; Department of Medical Humanities, Free University medical centre (VUmc), EMGO+ (Quality of Care), Amsterdam, The Netherlands

**Keywords:** Ethical challenges, Clinical ethics support service, Mental health care, Constructive disagreement, Ethics reflection group, Moral case deliberation, Coercion, Ethics

## Abstract

**Background:**

Little is known about how health care professionals deal with ethical challenges in mental health care, especially when not making use of a formal ethics support service. Understanding this is important in order to be able to support the professionals, to improve the quality of care, and to know in which way future ethics support services might be helpful.

**Methods:**

Within a project on ethics, coercion and psychiatry, we executed a focus group interview study at seven departments with 65 health care professionals and managers. We performed a systematic and open qualitative analysis focusing on the question: ‘*How do health care professionals deal with ethical challenges?*’ We deliberately did not present a fixed definition or theory of ethical challenge.

**Results:**

We categorized relevant topics into three subthemes: 1) Identification and presence of ethical challenges; 2) What do the participants actually do when dealing with an ethical challenge?; and 3) The significance of facing ethical challenges.

Results varied from dealing with ethical challenges every day and appreciating it as a positive part of working in mental health care, to experiencing ethical challenges as paralyzing burdens that cause a lot of stress and hinder constructive team cooperation. Some participants reported that they do not have the time and that they lack a specific methodology. Quite often, informal and retrospective ad-hoc meetings in small teams were organized. Participants struggled with what makes a challenge an *ethical* challenge and whether it differs from a professional challenge. When dealing with ethical challenges, a number of participants experienced difficulties handling disagreement in a constructive way. Furthermore, some participants plead for more attention for underlying intentions and justifications of treatment decisions.

**Conclusions:**

The interviewed health care professionals dealt with ethical challenges in many different ways, often in an informal, implicit and reactive manner. This study revealed nine different categories of what health care professionals implicitly or explicitly conceive as ‘ethical challenges’. Future research should focus on how ethics support services, such as ethics reflection groups or moral case deliberation, can be of help with respect to dealing with ethical challenges and value disagreements in a constructive way.

## Background

Working in mental health care entails experiencing typical ethical challenges which are related to its specific characteristics [1–5]. For example, in mental health care the epistemological distinction between what is considered as ‘illness’ and ‘health’ or ‘normal’ and ‘abnormal’ is often not as clear as in somatic health care [6]. Ethical challenges may emerge because some patients lack decisional capacity or because there is uncertainty or disagreement about that. The fact that some patients are admitted against their will, and that health care professionals are legally allowed to make use of coercive measures, also causes many different ethical challenges. Finally, limited evidence of whether coercive measures are ‘helpful’ or ‘effective’ increases the need for moral deliberation [7–11].

Dealing with ethical challenges in an appropriate way is important for several reasons. In the first place, we assume that paying attention to ethical challenges involves paying attention to defining and improving the quality of care. Furthermore, not paying attention to ethical challenges can be detrimental to patients, relatives, health care professionals, management; it might both challenge their cooperation and diminish the quality of the decision-making processes. Some studies report that not paying attention to ethical challenges might lead to increased ‘moral distress,’ which again might contribute to higher turn-over rates and sickness-rates among health care professionals [12]. Finally, recent evaluation research reports that health care professionals in mental health care highly appreciate dealing with ethical challenges using specific ethics support mechanisms such as moral case deliberation; it (in)directly improved their moral competence, the team cooperation, and the quality of care [13–16].

Although there is an increasing amount of literature on ethical challenges in health care [17, 18], most of the papers focus on the content of the ethical challenge itself, or present a predefined list of possible ethical issues to the respondents [19–22]. We found little information on how health care professionals actually *deal* with ethical challenges in health care [23–25]. This is in line with what Hurst and colleagues report [26], p.7: ‘*There has been no systematic, empirical examination of the values or the strategies actually employed by physicians to deal with the ethically problematic situations they face without help from ethics committees or consultants.*’ In the study of Hurst and colleagues [26], American internists, oncologists, and intensive care specialists were asked (by means of short telephone interviews) what kinds of strategies and approaches they used when facing ‘a recent ethical dilemma’. The physicians ‘*tried to obtain assistance, avoid conflict, and protect the integrity of their conscience and their reputation, as well as the integrity of the group of individuals participating in the discussion*’ [26], p.12. In a recent study in primary health care, Lillemoen and Pedersen [27] found that employees often deal with ethical challenges via informal discussions among colleagues, and discussions in various types of regular meetings at the unit. Quite a few respondents also reported that ethical challenges are often not discussed, left to the individual, or that their opinion has little importance. With respect to the specific domain of mental health care, we found only a few papers. Most of them focus mainly on dealing with ethical challenges within the pre-structured context of an ethics support service, such as moral case deliberation or ethics rounds [13–16, 28, 29].

Knowing how health care professionals deal with ethical challenges in mental health care when not making use of an ethics support service is important. It indirectly informs us about the relevance and significance of dealing with ethical challenges within the specific context of mental health care. It also might reveal expressed and non-expressed needs for ethics education, ethics training or ethics support. Furthermore, it is a necessary requirement in order to know how future ethics support services might fit the specific characteristics and needs of stakeholders and context within mental health care [17, 30, 31]. Finally, it can stimulate reflection about what kind of ‘dealing with ethical challenges’ should get covered by ethics support services, and what kind of ‘dealing with ethical challenges’ can get supported via more implicit support mechanisms within regular clinical practice [32].

The aim of this paper is therefore to inform health care professionals, managers, and those who aim to start with ethics support services in mental health care about how health care professionals deal with ethical challenges in situations where there is no explicit use of an ethics support service.

Our research question was: *How do health care professionals in mental health care deal with ethical challenges related to the use of coercion?*

## Methods

During the fall of 2012, two authors (MHH & BM) conducted seven focus groups interviews in seven wards in three mental health care institutions from different clinical fields (acute wards, rehabilitation unit, youth mental health care, geriatric mental health care, outpatient services). These interviews took place prior to the start of a two-year subproject on the implementation and evaluation of ethics reflection groups in mental health care, focusing on the use of coercion. This subproject was part of a larger project on ethics in mental health care.

The participants (in total 65), consisted of various health care professionals such as: nurses, nursing assistants, social workers, psychiatrist, psychologists, physicians, team leaders and management. Participants were invited to have a dialogue together and to express different or even opposite viewpoints. As moderators in the focus group interviews, we tried to stimulate an open dialogue and a safe atmosphere so that the participants could not only express their viewpoints but also could actually exchange and reflect upon their viewpoints together [33–35]. The focus group interviews lasted approximately 1,5 to 2 hours each, and were audio-taped and transcribed into 200 pages. Participants were asked to not mention specific patient names. In addition, all names within the transcripts were changed into fictitious names and the content was checked in order to guarantee privacy and confidentiality.

The focus group interviews were semi structured by three central questions:

1. What kind of ethical challenges related to the use of coercion do you experience?
2. How do you deal with these ethical challenges?
3. What do you expect from the ethics reflection groups?

This paper focuses on the answers to the second question. We informed the participants that they could use a broad understanding of coercion (i.e. formal, informal and perceived coercion). The analysis of the answers to the first research question are presented elsewhere [22]. We purposely only generally described ‘ethical challenge’ by using words like ‘ethical dilemmas’, ‘ethical problems’, ‘ethical issues’, ‘situations that caused a discussion or disagreement within the team or where you were concerned or uncertain about the right use of coercion’ or ‘difficult situations’. We wanted to know how health care professionals and leaders interpreted those terms without using specific moral theories or definitions of ethical challenges as the starting point for the interviews. In order to avoid general statements or opinions, we asked them to describe concrete and detailed situations in which they dealt with ethical challenges. After their initial answers to the second question we asked follow-up questions such as: What did you do? Who participated? Did you use a specific method?

### Analysis

The analysis of the transcribed interviews went through four phases. Through an initial open reading of the interviews each author presented separately and independently some preliminary topics. This was a hermeneutic and creative analysis attempting to grasp the main topics emerging from the rough data [36–38]. In the second phase, through a deliberative process among the authors, the initial topics were redefined into some main topics until consensus among the authors was reached. The consensus was based on the authors’ understanding of the words and expressions of the participants. In this process among the authors, some of the initial topics were excluded based on the authors’ preliminary understandings of ‘ethical challenge’ and ‘dealing with.’ Some of these examples will be discussed in the Discussion section. In the third phase, the first author (BM) re-read the transcripts of the interviews, checked, and if necessary, adjusted the main topics with examples and citations. Preliminary results and conclusions were thoroughly discussed with the co-authors and shared with other researchers during international workshops and expert seminars. Within the citations, we took out spoken language phrases such as ‘eh’ and ‘hmm’. All authors read through several drafts of this paper, including the final, in order to scrutinize the findings in the results section and control for haphazard or unbalanced used of citations and representations of the interviews [36].

### Research ethics

This focus group interview study has been approved by the Norwegian Social Science Date Services (approval September 17, 2012, project number 31360). All participants in the focus group interviews received an information letter beforehand in which we emphasized the voluntariness of their participation, their possibility to withdraw from the interview without giving reasons, the anonymity of the data, and the way we will archive and eventually destroy the research data. All participants gave their oral informed consent after having received written and oral information about the project. This information was given again when the interview began. We as authors report that there is no conflict of interest in publishing these data.

## Results

In the following we will present a systematic analysis of how the participants deal with ethical challenges related to coercion. We categorized the results into the following themes: 1) Identification and presence of ethical challenges; 2) What do the participants actually do when dealing with an ethical challenge?; and 3) The significance of facing ethical challenges.

### I. Identification and presence of ethical challenges

Most of the health care professionals seemed to have a general understanding of an ‘ethical challenge’ since they started to give descriptions of many different situations right away. They were able to present a broad variety of rich descriptions of situations in which they experienced either small or big ethical challenges [22]. In their expressions they often used words like ‘problem’, ‘dilemma’, ‘emotion’, ‘discussion’, ‘reflection’, or ‘thinking around.’

At the same time, some participants reported that they do not specify something as an *ethical* challenge in particular: ‘*We do not say: now this is ethics’*. Sometimes ethical issues are just presented as regular problems that are being discussed: ‘*Nurses discuss this, not as an ethical problem, but just as a problem.*’

The fact that ethical problems were not discussed as such, was not seen as problematic by some of the participants, while others explicitly wondered and struggled with how to distinguish between an ethical and a professional challenge: ‘*Is this ethical or is this professional? Or both?*’ Some reflected further upon whether it makes sense to distinguish the two: ‘*This is one of the reasons I'm struggling a bit with, with this here, that you should, what should we say, extract ethics from the rest of what we do. What is it we do then?*’

The presence of ethical challenges varied considerably. It seemed to have to do with how they understand what it means to deal with an ethical challenge. For example, some participants said they deal with ethical challenges every day: ‘*Yes, as a matter of fact, I think we discuss ethics every day. More or less, yes. What do we do now? Is this right?*’ Others said that basically it is all ‘ethical reflections’: ‘*I think all we do is ethical reflections, that is essentially what our job is.*’ In these citations, participants seemed to perceive questioning whether you are doing the right thing as a way of dealing with ethical challenges. Other participants did not refer to everyday situations or questions, but described in more detail the history of a single dramatic and often burdensome situation. The burden was sometimes related to the actual enforcement of coercive measures, to the possible negative consequences of their decisions, or to the consequences of an ethical challenge within the team (for example in cases of conflicts due to disagreements in the team). We will elaborate more on these experiences in the third part of the results section (‘*The significance of facing an ethical challenge*’).

Quite many participants mentioned that they do not succeed in paying explicit or enough attention to ethical challenges: ‘*We debrief and talk it through in order to make sure we follow the law as much as possible, but it is less of an ethical reflection.*’ ‘*So it* [i.e. discussing ethical issues; authors] *slips away in everyday situations*’. Some of them said that they should do it every day, the whole year, but that they lack the time to do so.

### II. What do the participants actually do when dealing with an ethical challenge?

In this section we will present what the participants of the focus group interviews actually do when dealing with an ethical challenge.

#### Individual reflection or with close colleagues

A considerable number of participants mentioned that they deal with ethical challenges alone. Dealing with ethical challenges is then seen as something you usually do on your own, without having a dialogue with others. One participant described an individual process in which she was pondering over the things one does.

*‘I think that ethical reflection…and I think that the formal, now we are sitting down to discuss, is in many ways one thing, but I think that everyone has some ethical reflection in between other tasks in the course of a day… even if we don’t sit down and think in bullet points or anything. You wonder about situations you’ve been in, and things you do. So even if it isn’t in any way formalized, you are doing it, throughout the day.’*

Participants mentioned that when they spoke with others about ethical challenges, quite often it was informal and with colleagues who have the same professional background (for example nurses with nurses). Some reported that there was little multidisciplinary exchange when it came to dealing with ethical challenges, especially between psychologists, psychiatrists, and doctors on the one hand, and nurses and social workers on the other.

#### Making use of regular or ad-hoc meetings

The health care professionals enumerated various types of meetings in which ethical challenges are discussed. Quite often it concerned regular meetings such as debriefing meetings in between two shifts, or the meetings in which the treatment team discusses the treatment plans of the patients. Other types of regular meetings are the supervision sessions, or the times where some educational activities are planned. However, ethical challenges are also discussed in informal meetings or small gatherings during the day: ‘*We discuss these kinds of issues in small groups, in the back room.*’

Quite often, participants mentioned that they arrange an ad-hoc meeting when experiencing an actual ethical challenge. This can be either during the process of experiencing an ethical challenge or retrospectively, when the situation that caused the ethical challenge is over. These meetings were often called ad-hoc meetings, reflection meetings, or crisis-meetings. *‘Those who were in the situation try to take a few minutes, then and there, in order to go through what happened.’* Participants did not mention prospective or structural meetings in which ethical challenges are discussed pro-actively.

In one focus group interview, they talked about four consecutive meetings with different participants. It concerned a situation in which two groups within a team disagreed in a not so constructive way about the coercion that one group had used.

***(I)****‘The first thing we did was reflect upon the situation itself, as it emerged that day, with the people involved: the doctor and about five nurses. We sat down and talked for quite a long time, actually. The doctor explained the reasons for the restraints in particular. And we could sort of bring in our own opinions about the situation that occurred and such. And some had, in retrospect, felt that the situation had been difficult, painful, and had felt very pressured. So she* [one of the nurses who felt very pressured, authors] *had had many difficult days at work’.*

***(II)****‘And so I have had several conversations with her* [that nurse, authors] *afterwards where I have discussed this particular situation, gone through what could of been done differently, whether there was another way we could have solved it, what she thinks about future situations’.*

***(III)****‘Yes, and then we have had a meeting with the person* [a colleague from another unit, authors] *who in a way was very opposed to the situation’.*

***(IV)****‘And we also reviewed it in the morning report the next day, more generally, not getting into all the details of the situation itself’.*

#### Use of explicit ethics meetings or explicit methods

None of the participants mentioned that they organized explicit ethics meeting such as an ethics reflection group or that they were using a method with an explicit focus on ethics. One physician, however, spoke favourably of bringing a case to an ethics committee. This happened when he was working at another workplace in another hospital. One participant talked about arranging special meetings: ‘*So, one of the things we do is to arrange ad hoc meetings, usually, so that the discussion is concentrated in what we actually call reflection meetings, which are being used quite actively for the difficult issues which move us emotionally.*’

Another participant talked about dealing with ethics in an explicit way. He mentioned that they sometimes identified basic principles and that they tried to weigh different principles against one another. ‘*So, what is it we should emphasize, should we in a way emphasize safety and the risk principle, or should we in a way emphasize the being-able-to-grow principle and autonomy so the patient can actually have a chance at self-development.* ‘Some said they attempt to find a balance between the legal and the ethical.

At the same time, most participants reported that they do not use a specific method. Some acknowledged that they are not very good at using a method at all. They mentioned that they need a specific kind of support in order to deal with ethical challenges. ‘*We are professionals, but we haven’t used a guide, or a method to do it.*’ ‘*So in a way we need guidance. Yes, how do we solve that. We probably are not very good at that.*’ ‘*When you have a good method, you probably hear more from more people than when you use a bad method, where you just sit and talk, and those who talk first also talk most*’.

#### The importance of discussing underlying intentions and justifications

Quite a few participants talked in various ways about the importance of knowing and understanding the underlying intentions and justifications of the use of coercion. Especially for those who work on a daily basis with coercion. He thought that more written information about the intentions behind the coercive actions will make it easier to understand why the coercive actions are planned or how they should be executed.

*‘I think that maybe the treatment plan has too many concrete actions, without the thought behind it. And that maybe could be traded in for a little sentence about intentions that could, yes, explain a little more about why and how*’.

Another participant presupposed that it will become easier when justifications for the use of coercion are more often discussed.

*‘The reason why I asked how often you discuss the justifications for the possible use of coercion is because I think it’s easier to take part in situations that feel ethically challenging if you understand their background’.*

Another participant pointed at the way the discussion among the various team members is organized, or not organized. She referred to the fact that those who have to work with coercion on the shop floor on a daily basis (e.g. nurses) often do not take part in the treatment meetings where the rationale for the use of coercion is being discussed.

*‘But if it is only done at a treatment meeting that occurs at random times with a handful of people present, and not those who might have to do these things in their daily practice, I would think you risk that it feels more difficult.’*

Another remark relates to the fact that it is difficult in practise to process and communicate all the ongoing changes in the treatment plan.

‘*And then in a way, when it needs to be communicated to many people over many days, and for instance temporary employees coming in, not because they aren’t good, but because they have not been part of the process, and execute it in a way that maybe was not the intention. I mean, they do what the treatment plan asks, but have in a way lost the reasoning behind it. That too can be very difficult, I think, and I don’t know how to handle it when you have all these people and changes nearly every day.’*

#### Dealing with ethical challenges due to disagreement

Ethical challenges emerged when health care professionals did not agree due to different viewpoints. Often disagreement involved a conflict between different values that are aimed for. However, not every disagreement automatically has to lead to an ethical challenge. There is quite some variety in the way health care professionals dealt with disagreement within their team. For example, one participant responded with curiosity when different viewpoints emerge. He thought it could be exciting to explore differences in ethical thinking and welcomed discussing them.

‘*I don’t really know how you think, I mean your ethical thought process, if it matches mine or not. So we don’t really know, and then we believe, and predict that we are at about the same level, but I don’t think we really are. What I think is that there are many differences. And it’s exciting to start that kind of discussion, really, because we might be at different levels, we haven’t looked at it close enough’.*

Another participant also stressed that dealing with ethical challenges based on disagreements can be useful in itself, as long as they are professional discussions, and as long as they focus on the patients. This specific focus makes the discussions good.

*‘And we have discussed it a lot, and I guess we have disagreed too, about what to think and what to do*… *But that is because we focus on the patient, and therefore we have good discussions too, even professional discussions, about what we actually believe and think about the patients and their treatment, and that is useful. Yes.’*

However, more often, dealing with ethical challenges due to disagreement became problematic for the participants. This was especially the case when disagreement or having different viewpoints on what was right to do, resulted in not only criticizing the coercive measure itself, but also the team as such. One participant described a situation in which coercion had been used. The involved professional said that she felt they had done a’*great job for this patient, even if it in a way was very uncomfortable to hold her down.*’ It seemed that the biggest ethical challenge was not the fact that they disagreed, but the way in which they communicated or did not communicate their disagreement with each other.

‘*I guess it was the kind of situation where the people who came afterwards weren’t able to see that this* [the use of coercion, authors] *was necessary, and therefore there were some reactions.’*

*‘I think that what became difficult after the situation had occurred, was that those who came later were very critical and puzzled about the doctor’s decisions and our handling of the situation that they were not part of. They felt that what we performed was abuse, and they had many opinions about it. And there was talk on other units about how we, on our unit, worked with patients. And how horrible it was to be a patient at our unit. And I think that those kinds of things are quite serious. I think that it is very important for those of us working on a psychosis unit that we have an unambiguous relation to the use of coercion.’*

In the last sentence of the above citation, it looks as if another possible dimension of the disagreement in this situation arises. It seems that the person is referring to an implicit norm: criticizing the use of coercion, when working at this unit, is in itself a problem. As if she/he is saying: if you work here, you should accept the use of coercion. If that is the case, then destructively dealing with disagreement might not be the problem, but disagreement in itself is perceived as being problematic.

#### How to deal with loyalty dilemmas?

Loyalty dilemmas were somehow related to dealing with disagreement, but in a slightly different way. Some participants talked about a dilemma between holding on to one’s own personal view, and at the same time acknowledging that it is important that a team responds in the same way to patients. If you really think what the team has agreed upon is wrong, and there is an implicit or explicit rule that everybody should do the same, then a dilemma related to loyalty seems inescapable.

‘*Yes, I mean, I can tell it does something to me, because me, as I am, thinks this isn’t right. When the staff decides that we should all do the same, I think it is important for me to do that, and so we do. That is what we are trying for him* [the patient, authors] *now. But for me, it is wrong, so I’m actually doing something to him that I think is completely wrong. And I can tell that it does something to me, yes. I often think it’s like that.*’

Saying that all team members should do the same can be both a strategy that tries to avoid ethical challenges and at the same time creates ethical challenges.

#### Responding to the message or to the messenger?

Sometimes asking questions and asking for argumentation (e.g. for a certain treatment policy) was interpreted by others as a sign of something else then asking for a justification. In one situation, the fact that a health care professional asked for clarification and reasons was interpreted by the chief physician as ‘identification with patients’ or as ‘protesting.’

Unit leader: ‘*Then there is nagging to those who make the decisions, like the chief physician, saying: Can you be more clear about your rationale? Can you justify? Can you repeat one more time why it is so important to go through with this here, so that those involved* [colleagues, authors] *get the information?’*

Chief physician: ‘*It’s not always coincidental who starts to identify with patients, and finds this* [the use of coercion, authors] *to be impossible or difficult. It is not seldom connected to other things than only this specific situation. There can be many additional reasons* [for nagging those who make the decisions, authors]*. Sometimes it can be that they protest, and at other moments it can be that they have their own specific reasons.*’

This fragment seems to refer to an important aspect of how team members interpret the question, the questioner and the process of questioning when dealing with ethical challenges due to disagreement. Do the team members interpret the question as factual and neutral, in other words: literally, just as it is? Or do they see the question as a sign of something else, for example as distrust or as over-identification with the patient? Are they attempting to discuss the question raised, or are they responding to or criticizing the questioner? In this way, asking for a rationale when there is a disagreement can quickly become personal, if the focus changes from the question and topic raised to the messenger. In this way ethical issues can be reframed as or reduced to a personal or psychological issue.

### III. The significance of facing ethical challenges

What does it actually mean to face an ethical challenge? What is at stake when being confronted with an ethical challenge?

#### From a daily routine to a paralyzing burden

As mentioned in the first section (‘Identification and prevalence of dealing with ethical challenges’), some participants said they deal with ethical challenges every day; it is a regular thing that is inherently attached to working as a professional in mental health care. This kind of ‘dealing with’ seems to refer to the act of ‘wondering whether we do the right thing’ or ‘asking questions about what is right’. However, most citations refer to situations in which the participants, both individually and as a team, become paralyzed due to the heaviness of dealing with ethical challenges. We will describe some of these burdens in more detail below.

#### Taking risks with serious consequences

Some participants described facing ethical challenges as a dramatic and exhausting experience when the decision that has to be made involves a certain risk. For example, one participant described the ethical challenge of whether to give a suicidal patient permission to leave the ward or not.

#### Team cooperation is challenged

Several participants talked about poor team cooperation due to disagreement within the team about what is morally right. For example, some team members feel as if they are set up against one another. The disagreement seems to become personal, and the team became divided into two sub-teams. One participant said it was difficult to continue the dialogue among them: ‘*I think this is the situation that has challenged our cooperation the most within our ward over the last two years. Because we were sort of challenged to continue the dialogue, and not remain opposed to each other.*’

#### Feeling personally distrusted

One health care professional even felt as if they mistrusted or were suspicious of him for what he had done. He describes a situation in which he dealt with a patient in a different way than most of the team thought was right. ‘*Because I think perhaps I have felt that I was, what shall I say, almost suspected of accommodating him* [the patient, authors] *somewhat more than I think I thought did.*’

#### Feeling exhausted and leaving the job

In one interview participants from an acute ward explained that the burden of being confronted with dilemmas all the time is so heavy that it causes an extra high turn-over rate among specialists; employees become tired of the dilemmas and work for just a short time on the ward. ‘*Among specialists there is a very high turn-over rate. It is quite common that specialists on acute wards quickly get burned out because of all the dilemmas and all the difficult situations. Faster turn-over of specialists. Two-three years is about average. I don’t know how many chief physicians I have seen come and go, but there are many that soon get tired of all of this.*’

#### Struggling with ethical challenges is a necessity

So far we have reported most on the participants’ burden when facing ethical challenges. However, some participants stressed that struggling with ethical challenges is a necessity in order to make sure that the patients receive good treatment. Through the process of struggling the participants try to ensure that what they do is good enough: ‘*Then we should reflect, right? Then that is to ensure that we are doing good enough work.*’

One of the participants pointed out another positive meaning of the struggling. Struggling, she said, is somehow also a sign of involvement and ‘being dedicated to.’ This participant thought that struggling is also a positive and appropriate response when facing ethical challenges related to this type of coercion (i.e. fixed tube feeding). Not responding in such a way and executing this kind of coercion just mechanically, can even be dangerous.

‘*I have to say I am happy that people come to my* [from the unit leader, authors] *office and say something about how it* [the use of coercion, authors] *felt, and that they have had a reaction to it. Because I think that in itself is a dilemma: that the day we no longer react to it, we are in dangerous waters, because it is such a major intervention, so just remaining in that discomfort ensures that you don’t just trust blindly, or that you do not mechanically do it. We want dedicated and engaged people. That means that we have to continue to work in that discomfort when we execute that type of coercion.*’

#### The need to understand when executing coercion

Understanding and lack of understanding plays a significant role in what it means to face an ethical challenge. Participants stressed the need to understand why coercion is being used, and the subsequent need to accept the reasons for the use of coercion when facing an ethical challenge. This is in line with what has been mentioned above about the importance of discussing underlying intentions and justifications. This applies especially to health care professionals who do not make the formal decision to use coercion, but at the same time are expected to execute coercive measures. ‘*I have to be able to conclude that it is useful – the coercion we practice. … and that the alternative is not better.*’ It becomes difficult when one does not feel confident that the coercive act is good for the patient. ‘*Executing coercion when in some way you do not feel wholly convinced that it clearly contributes to patient’s beneficence, that is not a situation that’s always easy to be in.*’ ‘*It gets into people’s bodies over the years.*’

Not understanding why one has to execute a coercive measure can become very burdensome. One participant talks about social workers who cry because it is intellectually so difficult to understand why they need to initiate forced tube feeding. Quite often it is so stressful for them that they say: ‘*I cannot do this anymore, I do not want this.*’

*‘Now we do it* [forced tube-feeding, authors] *more in our treatment without the somatic aspects being as clear or acute. That does something to them* [social workers, authors]*. I think that is where the most cases of crying social workers in my office have come from. Because they take part in situations that intellectually are so hard to understand; why they have to do it, because they can’t see the big picture. … That has, as you say Ingunn, been very burdensome. There are many times that many social workers say ‘I won’t do it anymore. I don’t want to. I will need a good explanation. Give me a rationale good enough to make me do it, because this has such high costs for the patient and for me.’*

## Discussion

Most of the participants of the focus group interviews in mental health care seem to have had a general and implicit understanding of an ‘ethical challenge.’ They presented many different ethical challenges; the content of these ethical challenges are described elsewhere [22]. Some participants of the focus group interviews mentioned that they deal with ethical challenges related to coercion all day, as an ongoing activity, such as wondering whether they are doing the right thing. Others mentioned stressful ethical challenges due to for example the high risks that are connected to their behaviour and decisions (e.g. when making decisions about discharging somebody with suicidal thoughts). Quite a few participants described how disagreement about the use of coercive measures had been a true burden to individuals and whole teams, sometimes resulting in teams that split up and teams or individuals that feel personally criticized. A small number of the participants stated that struggling with ethical challenges is a good thing, or even a necessity, in order to ensure that health care professionals do the right thing. Struggling, they described, is also a sign of being a dedicated professional, of not treating ethical challenges as routine issues.

Participants mentioned lack of time, knowledge and a specific methodology for dealing with ethical challenges in a multidisciplinary context. It seems that dealing with ethical challenges often took place in a rather implicit way. With implicit we mean two things: a) they did not frame the issues explicitly as being an ethical or moral issue, and b) they do not use specific meetings or methods with an explicit focus on ethics. Although implicitly, quite a few ethical challenges were discussed several times. This raises the question whether discussing an ethical challenge just once but in an explicit way, for example within an ethics committee or during a moral case deliberation, can be more time efficient than discussing that ethical challenge informally and implicitly on several occasions. Another question is whether there should be a kind of ‘stepped care’ plan for dealing with ethical challenges: the more serious and structural a certain ethical challenge, the more explicit ways of dealing with ethical challenges might be useful. One participant made use of the ethics committee at his former workplace; every Norwegian hospital trust nowadays has a central ethics committee [39]. Two participants spoke generally about *how* they actually discuss or reflect upon the ethical challenges by weighing conflicting principles.

Most situations that were described by the participants when talking about ‘dealing with ethical challenges,’ appeared to be reactions to concrete ‘problems’, directly after the problem had occurred. In other words, most ‘dealing with’ activities seem to be reactive, retrospective and within a short time-span. No prospective situations were discussed. Participants rarely referred to the use of, or to the conflicts caused by, normative frameworks such as legislation, policy, professional guidelines, or codes of conduct. Patients and relatives are often key stakeholders in the situations that are described as ethical challenges, yet they rarely seem to be involved in the actual dealing with those ethical challenges.

Participants stressed the need for understanding the use of coercion. Interestingly, participants themselves gave three possible reasons for how ethical challenges emerged or became more difficult to deal with. First, written and oral communication often focused too much on the concrete actions and too little on the underlying intentions and justifications. Second, there was a lack of time to communicate everyday changes in treatment decisions to all the staff members. And third, those who have to deal with ethical challenges concerning coercion on a daily basis were often not participants of treatment meetings where views and decisions related to coercion are discussed and explained. We think that better organized and more timely communication about situations which might cause ethical challenges or upcoming disagreement and confusion within the team can decrease both the prevalence and the severity of the ethical challenge.

Ethical challenges caused by moral doubt or uncertainty, that is, not knowing what is right to do, were not mentioned that often in the interviews. Many ethical challenges were related to situations in which there was disagreement or conflict. These results are also found in other papers [40–43]. Disagreements can be a good indicator that divergence of values might be at stake [40]. For example with respect to different viewpoints on good care. Or when there is variety in viewpoints among individual team members, while at the same time some people think that everyone should act upon the same treatment plan or policy. Interestingly, this study showed that disagreement as such does not automatically lead to experiencing an ethical challenge or having problems with team cooperation. Some teams seemed to cope very well with disagreements, or even seem to cherish disagreements; it can be seen as a sign of good cooperation and staying focused on what is morally right to do. Other teams experienced less constructive ways of dealing with disagreement, for example by means of privatizing existing disagreements, or criticizing persons or teams instead of opinions or viewpoints. We know from a large research tradition that disagreement or conflict often causes (moral) stress [44–46]. Research from Kellermans et al. [47] indicates that teams with so-called ‘constructive confrontation norms’ (i.e. ‘the combination of open expression, disagreement and the avoidance of negative affect’ [47], p.122) reduce the likelihood of conflict and are likely to improve decision quality. In line with this, Schippers et al. [48, 49] found that good team reflexivity can improve the cooperation and the quality of the work. Our findings urge for future research on how teams can develop constructive disagreement styles, and in which way ethics support services, such as ethics reflection groups or moral case deliberation, can be helpful.

The open approach in the analyses also stimulated reflections among us as researchers about what we mean with ‘dealing with.’ For example, we asked ourselves whether just being aware of an ethical challenge should be understood as dealing with an ethical challenge. Furthermore, what do they actually do when ‘discussing’ an ethical challenge? Should ‘dealing with’ at least consist of identifying some value uncertainties or value conflicts, and some basic reasoning about it? Is it possible *not* to deal with ethical challenges, e.g. through neglecting them or not seeing them at all? There is obviously a wide and nuanced range of what health care professionals actually do, feel and mean when they say they are dealing with ethical challenges. We can envision a dealing-with continuum with on the one hand ‘recognizing’ or ‘mentioning’ an ethical challenge, and on the other, a structured moral case deliberation in which the participants step by step go through the processes of systematically analysing and reasoning together with a trained ethics facilitator. Future research on dealing with ethical challenges could explore more specifically the different ways in which one can deal with ethical challenges, and the needs of the health care professionals.

Another point for discussion relates to the basic question of what health care professionals consider to be an *ethical* challenge. In our study, health care professionals seemed to have a general understanding since they named a broad variety of challenges. However, the way they characterized an ethical challenge varied. As authors we distinguished the following nine categories of ethical challenges as used by the participants in the focus group interviews: a) unidentified or implicit ethical challenges described as ‘a problem’ or ‘a discussion’ about what is right or good; b) ‘professional’ challenges; c) situations with implicit or explicit value-issues that are emotionally challenging for the employees; d) having reflections or explicitly asking questions about what is right or good; e) dilemmas where principles such as safety versus patient autonomy are identified and weighed; f) finding a balance between the legal and the ethical; g) disagree about what is morally right; h) disrespectful handling of disagreements between persons or (sub) teams; and i) feeling stuck between staying loyal to a decision from the team or your supervisor, versus your own convictions about what is morally right. Within these categories some challenges were clearly focused on patient care while some were more focused on cooperation among professionals. Sometimes an ethical challenge started with experiencing different viewpoints on what good patient care should be and ended up with an additional ethical challenge with respect to how to deal with those different viewpoints in a respectful way.

Some explicitly mentioned that they never used the word ‘ethical.’ Quite a few health care professionals struggled with the difference between a professional challenge and an ethical challenge. They mentioned that they lack a clear understanding of what ‘ethical’ exactly means. This seems to be in line with what Pelto-Piri and colleagues [41], p. 51 reported after having analyzed ethics diaries from health care professionals in mental health care “… *staff members have an extremely wide interpretation of the concept of ‘ethical considerations’.”*

And how do researchers define an ethical challenge? How do they actually interpret fragments of qualitative data as being an ethical challenge? While analyzing the focus group interview transcripts, we, as authors, discussed an example of how to distinguish an ‘ethical’ challenge from a ‘non-ethical’ challenge. An emotionally dramatic experience does not automatically have to lead to an ethical challenge. For example, when health care professionals are using forced medication against the will of a patient, we acknowledge that this can without doubt be a very stressful and challenging situation for the involved health care professionals. However, this does not immediately imply that these health care professionals are also uncertain or disagree about whether using forced medication in this particular situation is morally right or good. Other researchers, such as Pelto-Piri and colleagues [41], p. 50, use another distinction when stating that “a *large part of all statements can rather be interpreted as an expression of ‘moral stress’ than as genuine ethical reflections.”* Pelto-Piri and colleagues do not further explain and explore this distinction. Donaldson and colleagues [40] asked medical students to write down a case which according to the students had an ethical interest. In their analysis, Donaldson and colleagues distinguished between ethical-philosophical, legal-regulatory and practical-operational aspects of the students’ cases. They write [40], p. 817–818: “*The ethical-philosophical group included accounts that explicitly used terms related to morality (e.g. what ought or should be done), ethical theory (e.g. the nature of a doctor’s duty to be honest or whether a good outcome would justify a decision), or mid-level ethical principles. Also included were cases that posed questions about distributive justice”.* A more pragmatic description comes from a recent paper from Lillemoen and Pedersen on ethical challenges in primary health care: ‘*Ethical challenges may arise when we cannot do what we think ought to be done, or when there is doubt or disagreement about what is right or wrong*’ [27], p. 99.

At this moment, we distinguish the following six categories of ethical challenges as experienced by health care professionals: a) sincerely asking oneself whether one does the right or good thing; b) not knowing what is the right thing to do; c) being uncertain or in doubt about what is the right or good thing to do; d) disagree about what is morally right or good to do; e) knowing what is right or good to do but not being able or allowed to do that; and f) feeling obligated or forced to do something which you think is morally wrong or bad. In another paper based on these focus group interviews, in which we describe the content of the actual ethical challenges related to the use of coercion, we elaborate more on the values that were at stake within these ethical challenges [22].

Further empirical and conceptual research with respect to what is and should be conceived as an ethical challenge, is still needed. This might not only increase the clarity and consistent use of the term ‘ethical challenge’, it might also help to strengthen the moral awareness and sensitivity of health care professionals in general. Furthermore, empirical and conceptual research might clarify what kind of challenges clinical *ethics* support services (such as moral case deliberation, ethics reflection groups or ethics committees) should deal with. For example, would a ‘stepped care’ plan, which informs us what kind of implicit and explicit support fits with what kind of ethical challenge, be of help? Finally, the empirical and conceptual understanding of what is and should being conceived as an ethical challenges will be helpful in developing more targeted training and tools for both health care professionals and staff members of clinical ethics support services.

### Strengths and limitations of this study

Both a limitation and strength of this study is that we, purposely, did not define beforehand what we meant by ‘an ethical challenge’ and ‘dealing with.’ We just asked and looked at what kind of stories came up in order to see how health care professionals, through their stories, implicitly or explicitly define what they consider to be ‘an ethical challenge’ and ‘dealing with.’ This resulted in a rich and varied harvest on what participants understood as ‘ethical challenges’ and ‘dealing with.’ We did not explicitly help the participants in increasing the clarity and consistency of the use of these concepts *during* the focus group interviews. However, later on within the larger study on evaluation and implementation of ethics reflection groups, we offered more clarity regarding the use of these concepts through our presentations on ethics (reflection groups) and through the training of health care professionals as facilitators of ethics reflections groups.

Another limitation and strength is that the participants were selected from wards that were about to start with ethics reflection groups. This could mean that there was already a specific kind of awareness of, and interest in, working with ethics; perhaps more than other wards in mental health care. Although the results are clearly related to the use of coercion in mental health care, we think the underlying mechanisms of dealing with ethical challenges can have a wider relevance. A clear limitation is that we only talked to them about (dealing with) ethical challenges. If we had had more time, and had used participatory observation at the wards, we could clearly have gained more differentiated data.

## Conclusions

An open analysis of how health care professionals in mental health care actually experience and deal with ethical challenges related to coercion resulted in a rich harvest, both theoretically and practically. We found a broad variety of ways of dealing with ethical challenges. Most of ‘the dealing’ takes place without an explicit ‘ethics’ arena, focus, or methodology. Dealing with ethical challenges within the team in a constructive way can particularly be a challenge, certainly when it involves or leads to disagreement. Interestingly, disagreement was sometimes seen as a positive necessity in order to cooperate well together and to find out how to reach a view on good care, while within some teams the disagreement lead to an additional ethical challenge with threatened the quality of trust and cooperation within the team. The findings of this focus group study seem to indicate that dealing with ethical challenges is an important and quite often a burdensome part of working in mental health care which requires more, and a more appropriate, attention.

The study also revealed nine different categories of what health care professionals implicitly or explicitly conceive as ‘ethical challenges’. Future empirical and conceptual research is needed in order to further clarify the concept and practice of ‘ethical challenge.’ This research could not only shed a light on the question which challenge is appropriate for which kind of clinical ethics support service but it could also inform us about the training and tools for both health care professionals and staff of ethics support services. Furthermore, future empirical research could enlighten if, and in which way, specific ethics arenas such as ethics reflection groups or moral case deliberation, can actually be of additional help when dealing with ethical challenges and value disagreements.

## Authors’ information

BM, MHH & RP work respectively as Associate Professor of Clinical Ethics, Postdoc-researcher and Professor-researcher at the Centre for Medical Ethics, Institute of Health and Society, Faculty of Medicine, University of Oslo, P.O. Box 1130, Blindern, NO-0318, Oslo, Norway.

BM also works as Associate Professor of Clinical Ethics at the Department of Medical Humanities, Free University medical centre (VUmc), EMGO+ (Quality of Care), Amsterdam, the Netherlands.
